# *Micromonospora zhangzhouensis* sp. nov., a Novel Actinobacterium Isolated from Mangrove Soil, Exerts a Cytotoxic Activity *in vitro*

**DOI:** 10.1038/s41598-020-60677-0

**Published:** 2020-03-03

**Authors:** Geyi Fu, Ruijun Wang, Jinglin Ding, Huan Qi, Zhe Zhao, Can Chen, Hui Zhang, Zhenglian Xue, Jidong Wang, Min Wu

**Affiliations:** 1 Ocean College, Zhejiang University, Zhoushan, China; 2 Zhoushan Tourism & Health College, Zhoushan, China; 3 Zhejiang Key Laboratory of Antifungal Drugs, Zhejiang Hisun Pharmaceutical Co., Ltd., Taizhou, China; 4 College of Life Sciences, Zhejiang University, Hangzhou, China; 5 College of Biochemical Engineering, Anhui Polytechnic University, Wuhu, China

**Keywords:** Bacterial toxins, Soil microbiology

## Abstract

A new bacterial strain, designated HM134^T^, was isolated from a sample of soil collected from a Chinese mangrove *Avicennia marina* forest. Assessed by a polyphasic approach, the taxonomy of strain HM134^T^ was found to be associated with a range of phylogenetic and chemotaxonomic properties consistent with the genus *Micromonospora*. Phylogenetic analysis based on the 16s rRNA gene sequence indicated that strain HM134^T^ formed a distinct lineage with the most closely related species, including *M*. *rifamycinica* AM105^T^, *M*. *wenchangensis* CCTCC AA 2012002^T^ and M. *mangrovi* 2803GPT1-18^T^. The ANI values between strain HM134^T^ and the reference strains ranged from 82.6% to 95.2%, which was below the standard criteria for classifying strains as the same species (96.5%). Strain HM134^T^ and related species shared in silico dDDH similarities values below the recommended 70% cut-off for the delineation of species (range from 25.7–62.6%). The DNA G+C content of strain HM134^T^ was 73.2 mol%. Analysis of phylogenetic, genomic, phenotypic and chemotaxonomic characteristics revealed that strain HM134^T^ is considered to represent a novel species of the genus *Micromonospora*, for which the name *M*. *zhangzhouensis* sp. nov. is proposed. The extract of strain HM134^T^ was demonstrated to exhibit cytotoxic activity against the human cancer cell lines HepG2, HCT-116 and A549. Active substance presented in the fermentation broth of strain HM134^T^ was isolated by bioassay-guided analysis and purified afterwards. A new derivative of diterpenoid was identified through electrospray ionizing mass spectrometry (MS) and nuclear magnetic resonance (NMR). The compound showed different cytotoxic activities against cancer cells, with the highest cytotoxicity against HCT-116, corresponding to IC_50_ value of 38.4 μg/mL.

## Introduction

The genus *Micromonospora*, which belongs to the family *Micromonosporaceae* was first proposed by Ørskov^1^ with *Micromonospora chalcea* as its type species. *Micromonospora* species are widely distributed in nature and thrive in different environments, such as sandstone, soil, water, plants, insects, root nodules and mangrove sediments^2–10^. The genus has long been known as a significant source of secondary metabolites with diverse chemical structures and biological activities and is second only to *Streptomyces* in this respect, synthesizing up to 740 different bioactive microbial metabolites^11^. Members of the genus *Micromonospora* are Gram-positive, aerobic, non-motile spores directly from substrate hyphae and lack aerial mycelia. In general, the pigments of their mycelia are in orange, red or brown color. The range of G+C content of the DNA was 71.1–73.8 mol%^12^.

Mangroves are a unique woody plant community inhabiting intertidal coasts between land and sea, covering approximately 60–75% of the world’s tropical and subtropical coastlines and featuring in its ecological environment and high productivity^13^. Given that mangrove ecosystems have high salinity, strong winds, extreme tides, high temperature, anaerobic soils and high muddiness characteristics^14^, utilization of the mangrove microorganism resource with the potential of producing bioactive metabolites has increasingly received attention. Previous studies provided evidence that mangrove soil contains rich populations of *Micromonosporae*^15^. Furthermore, several novel species of the genus *Micromonospora* have been identified from marine and mangrove environments lately, including *Micromonospora haikouensis*^16^, *Micromonospora rhizosphaerae*^5^, *Micromonospora zhanjiangensis*^17^, *Micromonospora fluostatini*^18^, *Micromonospora sediminis*^19^, *Micromonospora sonneratiae*^20^, *Micromonospora mangrovei*^21^ and *Micromonospora maritima*^22^. Strains representing species of the genus *Micromonospora* have been recognized as important sources of bioactive secondary metabolites: gentamicin, sagamicin, sisomicin, verdamicin, everninomicin, lupinacidins A-C, netamicin, tetrocracin A, diazepinomicin and yangpumicin^11,12,23,24^. These findings suggested that the genus *Micromonospora* should remain a focus of research for the discovery of new bioactive metabolites. In the present study, a novel strain of the genus *Micromonospora* collected from the rhizosphere soil of the mangrove in Fujian, China was discovered. The extract from this novel strain exerted antitumor activity, in addition the activity compound present in the extract was characterized.

## Results

### Phylogenetic and genomic analyses

**Figure 1:**
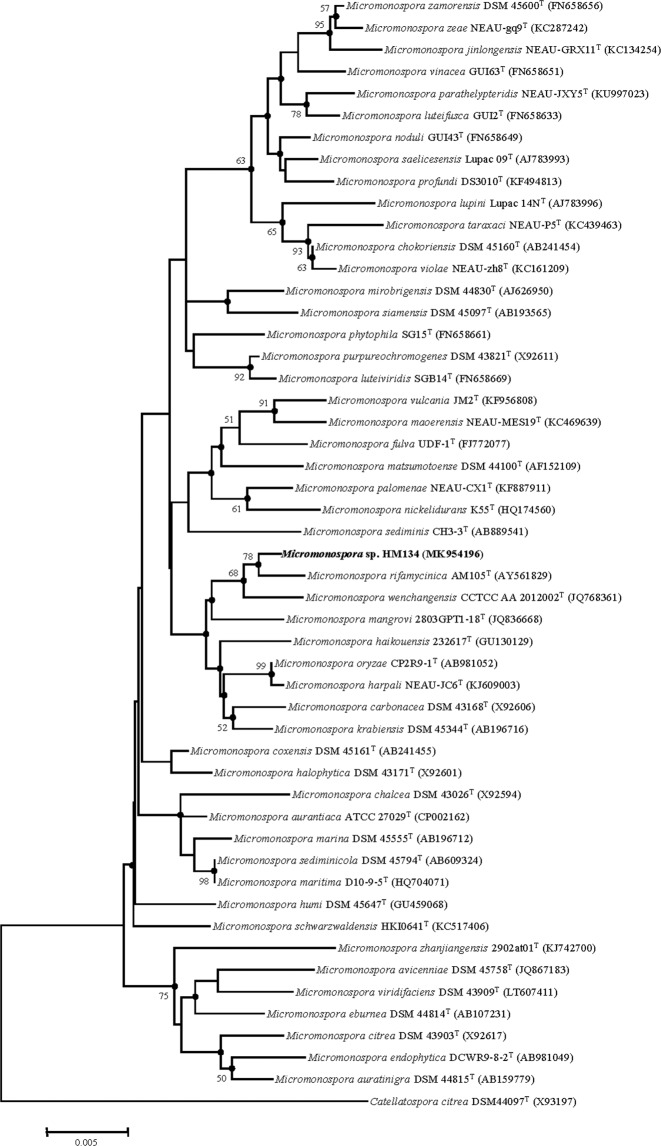
Neighbor-joining phylogenetic tree using the Kimura two-parameter model based on the 16S rRNA gene sequences of strain HM134^T^ and representatives of related taxa. Bootstrap values are based on 1000 replicates; values above 50% are shown. Filled circles indicate nodes also obtained in the maximum-likelihood and maximum-parsimony trees. Bar, 0.005 substitutions per nucleotide position.

**Figure 2:**
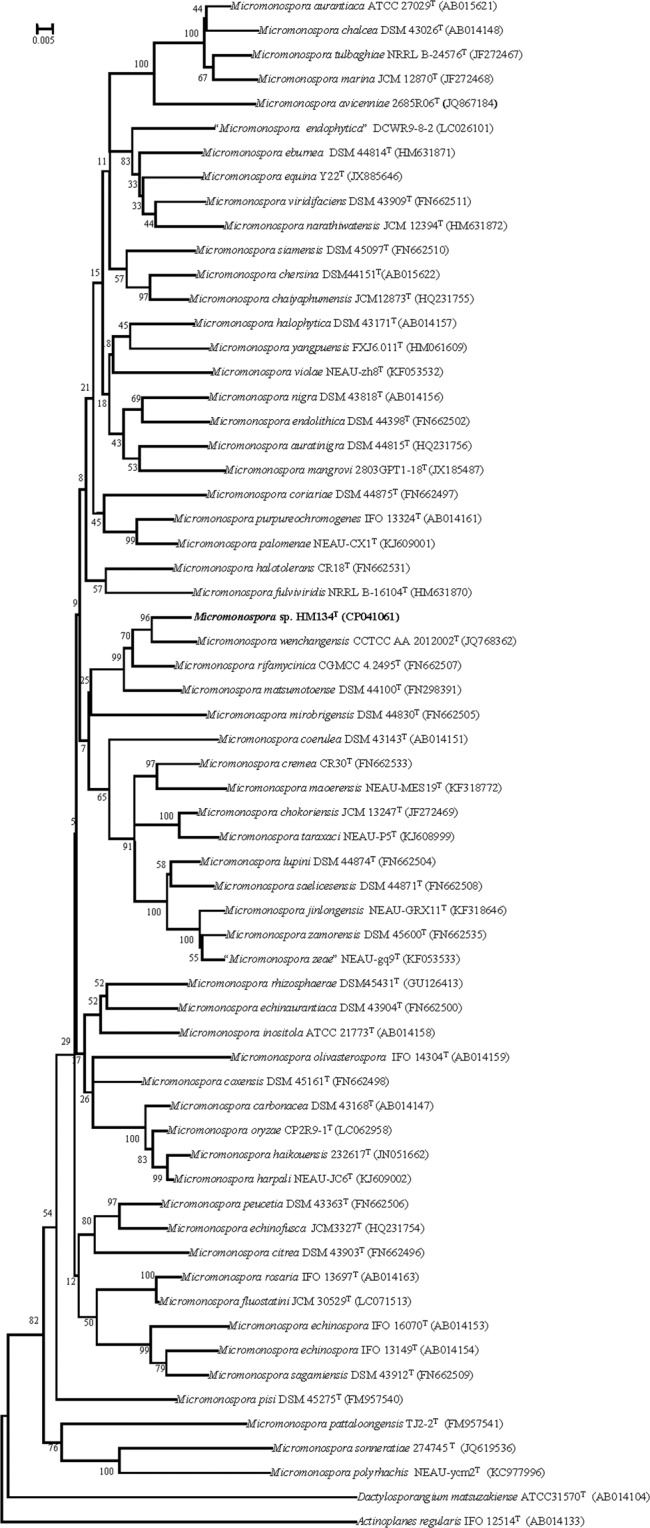
Neighbor-joining phylogenetic tree based on the partial *gyr*B gene sequences of strain HM134^T^ and representatives of related taxa. Bootstrap values are based on 1000 replicates; values above 50% are shown. Bar, 0.005 substitutions per nucleotide position.

The 16S rRNA gene sequence of strain HM134^T^ comprised 1480 nt. According to the analysis using the EzTaxon server, strain HM134^T^ was most closely related to *M*. *rifamycinica* AM105^T^ (99.6%), *M*. *wenchangensis* CCTCC AA 2012002^T^ (99.4%), *Micromonospora oryzae* CP2R9-1^T^ ^25^ (99.3%), *Micromonospora harpali* NEAU-JC6^T^ ^10^ (99.2%), *M*. *mangrove* 2803GPT1-18^T^ (99.1%), *Micromonospora krabiensis* DSM 45344^T^ ^26^ (99.0%), *M*. *carbonacea* DSM 43168^T^ ^27^ (99.0%), *M*. *haikouensis* 232617^T^ (99.0%), *Micromonospora schwarzwaldensis* HKI064^T^ ^28^ (99.0%), *Micromonospora sediminicola* SH2-13^T^ ^29^ (98.8%), *Micromonospora humi* DSM 45647^T^ ^30^ (98.8%), *M*. *maritima* D10-9-5^T^ (98.8%), *Micromonospora phytophila* SG15^T^ ^31^ (98.8%), *Micromonospora coxensis* DSM 45161^T^ ^31^ (98.8%) and shared less than 98.7% sequence similarity with the type strains of other species of the genus *Micromonospora*. The phylogenetic trees reconstructed based on the neighbor-joining (Fig. 1), maximum-parsimony (See Supplementary Fig. S1), maximum-likelihood (See Supplementary Fig. S2) methods and ARB program (See Supplementary Fig. S3). According to the neighbor-joining, maximum-parsimony and maximum-like methods, strain HM134^T^ stably formed a distinct lineage with the following related species: *M*. *rifamycinica* AM105^T^, *M*. *wenchangensis* CCTCC AA 2012002^T^ and *M*. *mangrovi* 2803GPT1-18^T^. The phylogentic tree using ARB program also indicated that strain HM134^T^ fell in the clade with *M*. *wenchangensis* CCTCC AA 2012002^T^ and *M*. *rifamycinica* AM105^T^. The DNA G+C content of strain HM134^T^ was 73.2 mol% (in silico), which is similar to those reported from most closely related species (71.0–71.7 mol %) and other strains in the *Micromonospora* genus. The complete genome sequence of strain HM134^T^ has been deposited at DDBJ/ENA/GenBank under the accession CP041061. The gene sequence *gyr*B was obtained from genome sequence (CP041061 from 7617 to 9563) and the phylogenetic tree based on *gyr*B gene sequence of HM134^T^ and other reference strains revealed that HM134^T^ should be placed in genus *Micromonospora* and most closely related to *M*. *wenchangensis* CCTCC AA 2012002^T^ (Fig. 2). The ANI values between strain HM134^T^ and additional reference strains were in the range 82.6–95.2% (See Supplementary Table S1), which was below the standard criteria for classifying strains as the same species (96.5%)^32^. Meanwhile, the in silico dDDH values between strain HM134^T^ and related species were range from 25.7–62.6%, which are below the recommended 70% cut-off for the delineation of species^12^ (See Supplementary Table S1), supporting that strain HM134^T^ can be considered to represent a novel species of genus *Micromonospora*.

### Chemotaxonomic analysis

The major respiratory quinone of strain HM134^T^ was identified as MK-10(H6). The absence of MK-10(H4), MK-9(H6) and MK-9(H4) could distinguish HM134^T^ from its close neighbor strains. The cell wall of strain HM134^T^ contained meso-diaminopimelic. Whole-cell hydrolysates predominantly contained xylose, arabinose and glucose. The detailed fatty acid and polar lipid profiles of strain HM134^T^ are shown in Supplementary Table S2 and Fig. S4, respectively. The most abundant fatty acids (>10%) detected in strain HM134^T^ included *iso*-C_16:0_ (30.3%), *iso*-C_15:0_ (14.1%) and 10-methyl C_18:0_ (TBSA, 12.4%), consistent with the previous findings that *iso*-C_16:0_ and *iso*-C_15:0_ were the predominant cellular fatty acids of the genus *Micromonospora*^33^ and reference strains (Supplementary Table S2). The polar lipid profile of strain HM134^T^ comprised diphosphatidylglycerol (DPG), phosphatidylethanolamine (PE), an unidentified phospholipid (PL1), three unidentified glycolipids (GL1, GL2 and GL3) and two unidentified lipids (L1 and L2). DPG and PE were also detected in the three reference strains (Table 1) and other *Micromonospora* species. The presence of GL1–3 and the absence of PI, PIM and PS could differentiate strain HM134^T^ from the reference strains.

**Table 1:** Differential phenotypic characteristics of strain HM134^T^ and the type strains of related species Strains: Strains: 1, HM134^T^; 2, *M*. *rifamycinica* AM105^T^; 3, *M*. *wenchangensis* CCTCC AA 2012002 ^T^; 4, *M*. *mangrovi* 2803GPT1-18^T^.

Characteristic	1	2	3	4
Temperature for growth (°C)	14–37	20–37^a^	16–45^b^	16–42^c^
pH range for growth	4.5–9.5	6–8^b^	5–9^b^	5.5–8^c^
NaCl tolerance (%)	7	4^b^	3^b^	3^c^
Nitrate reduced to nitrite	−	−	−	+
Gelatin liquefaction	+	+	+	−
Glucose fermentation	w	+	−	w
**Enzyme activity**				
Alkaline phosphatase	+	+	w	+
α-chymotrypsin	−	−	w	+
Esterase(C4)	w	−	−	−
Esterase lipase(C8)	w	w	−	−
α-galactosidase	−	−	−	w
β-glucosidase	w	+	w	w
Lipase(C14)	−	−	+	−
Naphthol AS-BI phosphohydrolase	−	−	−	w
N-acetyl-β-glucosaminidase	−	−	w	+
Urease	w	w	w	−
Valin arylamidase	w	−	w	−
**Hydrolysis of:**				
Starch	+	−	+	w
Tween 20	−	−	+	+
Tween 60	+	w	+	+
Tween 80	−	−	+	−
Esculin	+	+	+	w
**Utilization of:**				
N-Acetyl-glucosamine	w	+	w	w
D-Mannitol	w	+	−	w
Potassium gluconate	+	+	w	+
Malic acid	w	+	−	+
Major Polar Lipids	PE, DPG	PE, DPG, PIM, PS^c^	PE, DPG, PI, PIM^b^	PE, DPG, PI^c^
Major menaquinones	MK-10(H6)	MK-10(H6), MK-10(H4), MK-9(H4)^d^	MK-10(H6), MK-9(H6)^b^	MK-10(H6), MK-9(H6), MK-9(H4)^c^
DNA G+C content (mol%)	73.2	71.0^a^	71.7^b^	71.2^c^

All data are obtained from this study unless otherwise indicated. +, Positive; −, negative; w, weakly positive.

a:^79^. b:^80^. c^21^. d:^16^.

### Phenotypic Characterization of the HM134 Isolate

Strain HM134^T^ grew well on ISP 1, ISP 2, ISP 3^34^ agars, tryptone soy agar and nutrient agar after 7–14 days at 28 °C, moderately on ISP 4, ISP 5^34^ and Streptomyces agar, and poor on ISP 7^34^ and calcium malate agar^35^. The colors and substrate mycelia were dependent on the culture medium used (Table 2). Aerial hyphae were absent and no soluble pigment was produced in any of the culture media. Morphological observation of strain HM134^T^ revealed that single spores were formed on the end of substrate hyphae (Supplementary Fig. S5). Growth was observed at pH 4.5–9.5 (optimum pH 5.5–8.5), with 0–7% NaCl tolerance (optimum 0–1%) and at 14–37 °C (optimum 20–28 °C). Nitrate was weakly reduced to nitrite but not to N_2_. Cells were found to be positive for catalase but negative for melanoid pigment production. Liquefaction of gelatin, milk coagulation, hydrolysis of esculin and soluble starch were found to be positive, but negative for hydrolysis of cellulose, H_2_S production, Voges-Proskauer test and methyl red test. According to Table 1, strain HM134^T^ and all the reference strains could hydrolyze esclin and gelatin, utilize N-acetyl-glucosamine and potassium gluconate, meanwhile esterase(C4) in strain HM134^T^ is weakly positive, which could be distinguished from other reference strains. Also the ability to hydrolyze starch revealed discrepancy between strain HM134^T^ and the most close strain *M*. *rifamycinica* AM105^T^. In addition, thirteen strains were chosen as representatives of adjacent clusters, the phenotypic characteristics were collected from literatures and compared with strain HM134^T^ (See Supplementary Table S3). The detailed physiological and biochemical properties are presented in the species description.

**Table 2:** Phenotypic characteristics of strain HM134^T^ on different mediums.

Medium	Growth	Color of colony	Diffused pigment	Colony surface
ISP1	Abundant	Beige	None	Rough
ISP2	Abundant	Beige	None	Rough
ISP3	Abundant	Deep orange	None	Rough
ISP4	Moderate	Oyster white	None	Smooth
ISP5	Moderate	Oyster white	None	Smooth
ISP7	Poor	Ivory	None	Smooth
Tryptone Soy agar	Abundant	Beige	None	Rough
Streptomyces agar	Moderate	Oyster white	None	Smooth
Calium Malate Agar	Poor	Oyster white	None	Smooth
Nutrient Agar	Abundant	Pastel yellow	None	Rough

All data were obtained from this study.

### Cytotoxic activity of strain HM134^T^ extract

**Figure 3:**
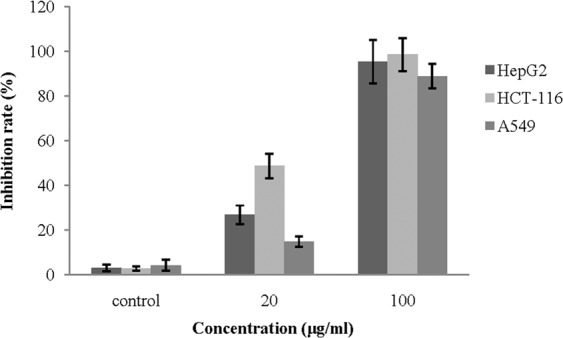
Cytotoxic activity of HM134^T^ extract against human cancer cell lines. The measurement of inhibition rate was done using CCK-8 method. The graphs show cytotoxicity effect of HCT-116 HepG2 and A549. Medium containing 0.5% dimethyl sulfoxide was used as a control. All data are presented as the mean ± standard deviation from three experiments.

The cytotoxic potential of HM134^T^ extract was tested against several human-derived cancer cell lines (HCT-116, HepG2 and A549) and the results are summarized in Fig. 3. All cancer cell lines showed susceptibility to the extract of stain HM134^T^ with inhibition ratios range from 88.84–98.5% (100 μg/mL extract was tested). The extract exhibited the highest toxicity against HCT-116 cells with the inhibition ratios of 98.50 ± 4.8% and 48.73 ± 2.5% when tested at the concentration of 100 μg/mL and 20 μg/mL, respectively. As indicated, the inhibition ratio of A549 was significantly reduced at lower extract concentration (decreased to 14.94 ± 2.3%). The correspongding inhibition ratios on HepG2 cells were 95.34 ± 5.7% and 26.84 ± 4.2%, respectively. Furthermore, we observed a dose-dependent effect when the extract was tested against human-derived cancer cell lines. Overall, the results suggested that the HM134^T^ extract has a higher cytotoxic effect against the HCT-116 cell lines than the HepG2 and A549 cell lines. Based on the results of the cytotoxicity, we further characterized the bioactive metabolites of strain HM134^T^ against HCT-116, HepG2 and A549 cells.

### Identification of active compounds

**Figure 4:**
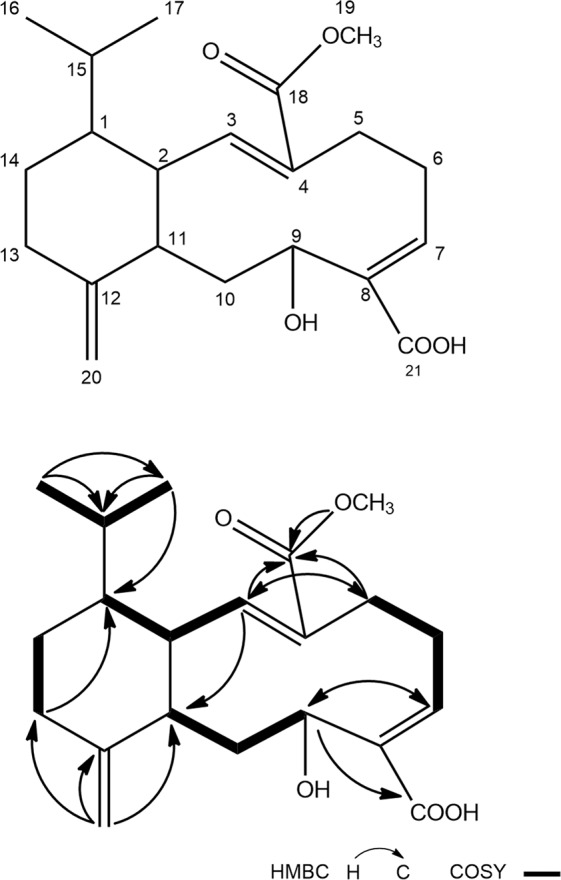
Structure of compounds **1** from strain HM134^T^.

Bioassay-guided isolation of the active components of HM134^T^ was carried out as described in the Materials and Methods. The active metabolite was characterized by spectroscopic analyses and by comparison with the data available from literature. This compound was obtained as a yellow amorphous gum. Its molecular formula, C_21_H_30_O_5_, was deduced from TOF-MS m/z 363.2182 [M+ H]^+^, which was consistent with the ^1^H NMR and ^13^C NMR data (See Supplementary Figs. S6–S13 and Table S4). As shown in Fig. 4, the structure of compound **1** displayed a novel diterpenoid derivative.

The IR spectrum showed characteristic absorptions for hydroxyl (3659 cm^−1^) and carbonyl (1704 cm^−1^). The ^1^H NMR spectrum of compound 1 indicated the presence of two aliphatic methyl doublets at *δ*_H_ 0.80 (3 H, d, *J* = 6.6 Hz) and 0.83 (3 H, d, *J* = 6.6 Hz), a methoxy at *δ*_H_ 3.75 (3 H, s), an oxygenated methane proton at *δ*_H_ 4.11 (1 H, dd, *J* = 10.6, 4.6 Hz), one olefinic methylene at *δ*_H_ 4.64 (1 H, br s),4.69 (1 H, br s), and two olefinic methines at *δ*_H_ 6.35 (1 H, t, *J* = 8.5 Hz), 6.91 (1 H, d, *J* = 12.4 Hz). The ^13^C NMR and HSQC spectra exhibited 21 carbon resonances, comprising two carboxyls (*δ*_C_ 168.6,170.0), three olefinic quaternary carbons (*δ*_C_ 128.4, 131.4, 150.0), two olefinic methines (*δ*_C_ 145.4, 145.4), one olefinic methylene (*δ*_C_ 109.2), one oxygenated methine (*δ*_C_ 78.7), four methines (*δ*_C_ 30.9, 41.8, 42.5, 46.5), five methylenes (*δ*_C_ 26.0, 26.9, 28.1, 33.0, 39.3), a methoxy (*δ*_C_ 51.8), and two methyls (*δ*_C_ 21.0, 21.5). Detailed analysis of the ^1^H-^1^H COSY and HMBC correlations (Fig. 4) established the structure of compound **1**. An isopropyl moiety was established by the ^1^H-^1^H COSY correlations of H_3_-16/H-15/H_3_-17 and the HMBC correlations from H_3_-16 to C-15 and C-17, from H_3_-17 to C-15 and C-16. Furthermore, ^1^H-^1^H COSY correlations of H-1/H-2/H-3, H_2_-5/H_2_-6/H-7, H-9/H_2_-10/H-11 and H_2_-13/H_2_-14 indicated the four structural moieties of C-1-C-3, C-5-C-7, C-9-C-11 and C-13-C-14. The HMBC correlations from H_2_-20 to C-11, C-12 and C-13 revealed the connection of C-11 and C-13 through C-12. The linkage of C-1 and C-14 was confirmed by the HMBC signal from H_2_-13 to C-1. The HMBC correlation of H-3 and C-11 established the connection of C-2 and C-11.The observed ^1^H-^13^C long-range correlations from H-7 to C-9, C-21 and from H-9 to C-7 and C-21 showed that C-7 and C-9 was connected through C-8 and a carboxyl group was situated at C-8. Similarly, HMBC correlations from H-3 to C-5 and C-18, from H_2_-5 to C-3 and C-18, from H_3_-19 to C-18 indicated that C-3 and C-5 was connected via C-4 and one acetate was attached to C-4. As a result, the structure of the compound was shown in Fig. 4. According to the NOESY data (See Supplementary Fig. S14), the correlation of H-2/H-5 and H-7/H-10 revealed that both the double bonds could be assigned to be of E-configuration. Compound **1** was named as (7*E*,11*E*)-6-hydroxy-1-isopropyl-11-(methoxycarbonyl)-4-methylene-1,2,3,4,4a,5,6,9,10,12a-decahydrobenzo[10]annulene-7-carboxylic acid.

### Cytotoxicity of bioactive metabolite from Strain HM134^T^

The cytotoxic activities of compound **1** against the HepG2, HCT-116 and A549 human cancer cell lines were measured. It suppressed the proliferation of HepG2, HCT-116 and A549 cancer cells. This compound exhibited the strongest cytotoxicity against HCT-116 cells, with the IC_50_ value of 38.4 μg/mL. Meanwhile, it also exhibited moderate cytotoxicity against HepG2 cells and A549 cells, with IC_50_ values of 69.8 and 50.5 μg/mL, respectively.

### Genotypic characterization of the HM134^T^ isolate and screening for antibiotic biosynthetic gene clusters

The genome of strain HM134^T^ consists of one circular chromosome (7,565,212 bp, 73.2% G+C), with the absence of plasmid. A total of 6853 protein-coding sequences (CDS) and 109 RNA genes were predicted. The genomic features of strain HM134^T^ were summarized in Table 3.

**Table 3:** General features of the genome of Micromonospora sp. HM134.

Attribute	Value
Size (bp)	7,565,212
DNA G+C content (%)	73.2
CDSs	6853
CDSs assigned to COGs (percentages)	4688 (68.4)
CDSs assigned to KEGG (percentages)	3061 (44.7)
CDSs assigned to GO (percentages)	3710 (54.1)
rRNA operon (16S-5S-23S rRNA)	12
tRNAs	53
CRISPR repeats	3

Among the 6853 CDSs, only 4688 CDSs were classified into COG categories (See Supplementary Table S5). The major categories included transcription (9.8%), carbonhydrate and amino acid transport and metabolism (8.2% and 7.7%, respectively), signal transduction mechanisms (5.9%), coenzyme, lipid and inorganic ion transport and metabolism (5.7%, 5.6% and 5.4% respectively). Noteworthy, the poorly characterized category General function prediction only (11.85%) included many secondary metabolites synthesis clusters, indicating that there were lots of functional genes were unclear in strain HM134^T^. A total of 32 secondary metabolite gene clusters were detected using antiSMASH. There were 4 terpene clusters, 2 type I PKS clusters, 1 type II PKS cluster, 1 type III PKS cluster, 5 NRPS clusters, 1 siderophore, 3 bacteriocins and 11 heterozygous PKS-NRPS clusters found in strain HM134^T^. These results highlighted the genomic potential of the inspected isolates for natural products discovery. Furthermore, the presence of terpene clusters is primarily responsible for the synthesis of the newly terpene derivate.

## Discussion

Based on the polyphasic approach analysis, strain HM134^T^ was markedly different from the most closely related type strains of the genus *Micromonospora*. Therefore, strain HM134^T^ merits assignment to a novel species in the genus *Micromonospora*, for which the name *M*. *zhangzhouensis* sp. nov. is proposed. The type strain is HM134^T^.

The extract from strain HM134^T^ demonstrated 88.8–98.5% inhibition ratio against human cancer cell lines (HepG2, HCT-116 and A549) when tested at a concentration at 100 μg/mL. Since the strain HM134^T^ is a novel *Micromonospora* species, it would be a potential reservoir of natural products with cytotoxic activity. Bioassay-guided separation and purification by multiple methods were successfully applied to identify the active fractions. The active compound **1** identified as a novel diterpenoid derivative that exhibited cytotoxic activity against cancer cells.

Terpenes are one of the major secondary metabolites with different compound types, including monoterpenenes, diterpenes, sesquiterpenes, triterpenes, sesterterpenes and norterpenes^36,37^. Although actinomycetes harbor the genetic potential to produce terpenes, terpenoid natural products are rarely observed when cultured in fermentation broths. The carbon skeleton of compound **1** is similar to cembrane-type diterpenoids, which form a large and structurally different group of natural products that can be isolated from both terrestrial and marine organisms. Cyclisation of a geranylgeraniol derived precursor between carbon 1 and 14 generates a 14-membered diterpenoid, named cembrane or thumbergane^38^. As previously reported, coelenterates are recognized as the most prominent source of cembranoids^39,40^. From a biomedical perspective, cytotoxicity is the most remarkable characteristic of cembranoids. In addition, cembranoids possess multiple biological activities such as neuroprotective, anti-inflammatory, antimicrobial, antiarthritic effects. In previous study, Luo, *et al*.^41^ reported that eight cembrane-type diterpenoids were isolated from *Macaranga pustulat*, *a* including three new compounds that exert cytotoxicity (IC_50_ > 20 μM) towards human cancer cell lines (CNE1, CNE2 and HCT116). In another study, researchers isolated two new cembrane diterpenes from the flowers of *Nicotiana tabacum* L. with anti-tumor activities against human tumor cell lines (HepG2, A549 and HCT-116)^42^. In addition, four unknown cembrane-type diterpenoids exhibiting hepatoprotective activity at 10 μM against paracetamol-induced HepG2 cell damage were isolated from the gum resin of *Boswellia sacra* Flueck^43^. Another similar skeleton structure of compound **1** is cladiellane-type diterpenoid, one of the class of metabolites from gorgonians with the skeletons containing an ether bridge across C-2 and C-9^44^. These metabolites also displayed a wide range of bioactivities. For example, Ru, *et al*.^45^ reported three cladiellane-type diterpenoids exhibiting moderate anti-inflammatory activity with the IC_50_ values range from 15.8 to 43.7 μM. Previous studies demonstrated that hydroxylated derivatives showed improved anti-cancer activity^46^, suggesting that the hydroxyl group of compound **1** may contribute to the suppressive effect on human cancer cells.

In summary, the strain HM134^T^, a novel species of the genus *Micromonospora* was successfully isolated from the mangrove soil of Zhangzhou, China. The findings of this study demonstrated that the strain HM134^T^ exhibited significant cytotoxic activity against human cancer cell lines (HepG2, HCT-116 and A549) and a new diterpenoid derivative was found. This study provides a comprehensive description of the novel strain *Micromonospora zhangzhouensis* HM134^T^ and elucidated the potential of the strain as a resource for anticancer or drug discovery. Hence, further studies to provide in-depth research on the cytotoxic property of this strain are highly valuable.

### Description of *Micromonospora* sp. nov

*Zhangzhouensis* (zhang.zhou.en′sis. N.L. fem. adj. *zhangzhouensis* referring to Zhangzhou, a city in Fujian, China, from where the type strain was isolated).

Cells are gram-positive, non-motile, actinomycete that forms well-developed and branched substrate hyphae. Oval spores are smooth-surfaced with the size of 0.6 × 0.8 μm. Colonies range from pastel orange to beige in corlor when cultured on ISP 2 medium. The colors of the substrate mycelia are dependent on the culture medium. Aerial hyphae were absent and no soluble pigment was produced in any of the culture media. Cells grow well on ISP 1, ISP 2, ISP 3 agars, tryptone soy agar and nutrient agar after 7–14 days at 28 °C, moderately on ISP 4, ISP 5 and Streptomyces agars, poorly on ISP 7 and calcium malate agar. Growth was observed at pH 4.5–9.5 (optimum pH 5.5–8.5), with 0–7% (w/v) NaCl tolerance (optimum 0–1%) and at 14–37 °C (optimum 20–28 °C). Nitrate was weakly reduced to nitrite. Nitrite was not reduced to N_2._ Cells were positive for catalase, alkaline phosphatase, acid phosphatase, β-galactosidase, α-glucosidase, leucine arylamidase, PNPG and trypsin. Weakly for arginine dihydrolase, esterase (C4), esterase lipase(C8), β-glucosidase and valine arylamidase. Negative for α-chymotrypsin, arginine dihydrolase, cystine arylamidase, α-fucosidase, α-galactosidase, β-glucuronidase, α-mannosidase, lipase (C14), napthol-AS-BI-phosphohydrolase and N-acetyl-β-glucosaminidase. Positive for liquefaction of gelatin, milk coagulation, hydrolysis of esculin and soluble starch, degradation of Tween 40 and Tween 60. Weakly for glucose fermentation. Negative for hydrolysis of cellulose, production of H_2_S, Voges-Proskauer test, methyl red test, degradation of Tween 20, Tween 80, adenine, guanine, xanthine and hypoxanthine. Positive for utilization of L-arabinose, D-glucose, maltose, D-mannose, potassium gluconate and adipic acid. Weakly utilize N-acetyl-b-glucosaminidase and D-mannitol, malic acid. Do not utilize n-capiric acid, citrate, phenylacetic acid or tryptophan. The major fatty acids (>10%) were *iso*-C_16:0_ (30.3%), *iso*-C_15:0_ (14.1%) and 10-methyl C_18:0_ (TBSA, 12.4%). The polar lipid profile of strain HM134^T^ comprised diphosphatidylglycerol, phosphatidylethanolamine, an unidentified phospholipid, three unidentified glycolipids and two unidentified lipids. The major respiratory quinine was MK-10(H6). The cell wall of strain HM134^T^ contained meso-DAP. Whole-cell hydrolysates predominantly contained xylose, arabinose and glucose. The type strain HM134^T^, was isolated from the rhizosphere soil of the mangrove in Fujian, China. The GenBank accession number for the 16S rRNA gene sequence and the genome sequence of strain HM134^T^ is MK954196 and CP041061, respectively. The DNA G+C content of the type strain was determined to be 73.2 mol% (in silico).

## Materials and Methods

### Sample collection and isolation of actinomycetes

Strain HM134^T^ was isolated from a soil sample collected in the mangrove *Avicennia marina* forest of Zhangzhou (N24°40′, E118°11′), Fujian Province (China) during the spring of 2015 and subsequently stored at 4 °C until use. The soil sample was suspended in sterile water and diluted in a tenfold series. The dilutions were then spread onto modified *Gauze’s No*.*1* agar^47^ supplemented with nystatin (50 μg/ml) and nalidixic acid (25 μg/ml) incubated at 28 °C for 14 days. After incubation, a yellow colony was selected and purified by repeated streaking on to *Gauze’s No*.*1* agar. The strain was routinely cultured in *Gauze’s No*.*1* agar and preserved as glycerol suspensions (20%, v/v) at −20 °C.

### DNA extraction and purification

Genomic DNA was extracted using a Quick Bacteria Genomic DNA Extraction kit (DongSheng Biotech). The 16S rRNA gene of strain HM134^T^ was amplified using the universal primers 27 F (5′-AGAGTTTGATCMTGGCTCAG-3′) and 1492 R (5′-TACGGYTACCTTGTTACGACTT-3′)^48^. The amplification products were cloned into the pMD19-T vector (TaKaRa) and then sequenced. The obtained 16S rRNA gene sequence (1480 nt) was analyzed by performing pairwise sequence alignments using the NCBI nr database (http://www.ncbi.nlm.nih.gov) and the EzTaxon-e server (https://www.ezbiocloud.net)^49^. Multiple sequence alignments based on the 16S rRNA gene sequences of strain HM134^T^ and related taxa were performed using the CLUSTAL X program of the MEGA 5 software package^50^. Phylogenetic trees were reconstructed from 1000 replicates using the neighbor-joining^51^, maximum-likelihood^52^ and maximum-parsimony^53^ methods based on 1000 replications and bootstrap analysis. To testify the phylogenetic tree reconstructed by MEGA 5, All-Species Living Tree LTPs123 and database arb-6.0.6 were used as the reference, SINA webserver^54^ and ARB software^55^ were used for alignment of 16S rRNA gene sequence into LTPs123 and generation of a new maximum-likehood phylogenetic tree, respectively.

The complete genome was sequenced at the Beijing Genome Institute (BGI, Shenzhen, China) using a PacBio RS II platform and Illumina HiSeq. 4000 platformand. The genome was assembled as described previously^56^. The DNA G+C content was determined by Rapid Annotation System Technology (RAST)^57^. The genome sequences of ten reference strains were retrieved from the GenBank database (Project accession numbers were listed in Supplementary Table S1). The average nucleotide identity (ANI) was calculated using the OrthoANI algorithm of the Chun lab’s online Average Nucleotide Identity calculator^58^. The in silico dDDH value was calculated using the GGDC web server^59^ available at https://ggdc.dsmz.de/ggdc.php#. Glimmer version 3.02 (Delcher *et al*., 2007) was used to predict open reading frames (ORFs) according to the manufacturers’ instructions. Following this, ORFs were annotated using the NCBI NR, SwissProt^60^, KEGG^61^, GO^62^ and COG^63^ databases. The tRNA and rRNA genes were predicted using tRNAscan-SE^64^ and RNAmmer^65^, respectively. CRISPR repeats were predicted using CRISPR finder^66^. Biosynthetic gene clusters of secondary metabolites were predicted using the antiSMASH 3.0 web server^67^. The gene sequence of *gyr*B was identified by RAST of the genome sequence of strain HM134^T^. The phylogenetic tree based on the housekeep gene (*gyr*B) of strain HM134^T^ and other strains was constructed using neighbor-joining method, with the Tamura-3-parameter model and G substitutions. *M*. *rifamycinica* AM105^T^, *M*. *wenchangensis* CCTCC AA 2012002 ^T^ and *M*. *mangrovi* 2803GPT1-18^T^ were obtained from CGMCC (China General Microbiological Culture Collection Center,) and CCTCC (China Center for Type Culture Collection). Unless otherwise stated, all strains were incubated in TSB at 28 °C.

### Phenotypic characterization of the HM134^T^ Isolate

The temperature range for growth was determined in ISP2 (pH 7.0) at 4–45 °C (4, 10, 15, 20, 28, 30, 35, 37 and 45 °C). The tolerance to NaCl concentrations was tested in ISP2 (pH 7.0) with the concentrations of NaCl at 0–10.0% (w/v), with an increment of 0.5%. The pH range for growth was tested with an interval of 0.5 pH unit, by supplementation of ISP 2 medium with 30 mM buffering agents at 28 °C: 2-(*N*-morpholino) ethanesulfonic acid for pH 5.5–6.5, 3-(*N*-morpholino) propanesulfonic acid for pH 6.5–8.0, tricine for pH 8.0–9.0, and bis-Tris propane for pH 9.0–9.5. The optimal growth was determined after 7 days of incubation, and the growth limits were determined after 14 days of incubation. HM134^T^ was incubated at 28 °C for 21 days on ISP 2 medium, and cell morphology was examined and observed using an optical microscopy (BX40; Olympus) after Gram staining, a transmission electron microscopy (80 kV, JEM-1230; Jeol) after uranyl acetate (0.5%, w/v) staining and a scan electron microscope (3.0 kV, SU8010, Hitachi) after fixation by osmium tetroxide vapor (4%, w/v). The culture characteristics were determined following growth on tryptone-yeast extract agar (ISP 1), yeast extract-malt extract agar (ISP 2), oatmeal agar (ISP 3), inorganic salts-starch agar (ISP 4), glycerol-asparagine agar (ISP 5), and tyrosine agar (ISP 7) agars^34^; *Gauze’s No*.*1* agar, nutrient agar, tryptone soya agar^68^ and calcium malate agar^35^ for 14 days at 28 °C. The colors of substrate and aerial mycelia were determined based on comparison with the ISCC-NBS color system^69^. Catalase and oxidase activity were tested following the method described by Sun, *et al*.^70^. Hydrolysis tests were performed with different substrates supplemented with gelatin, skimmed milk, starch (5 g/L); CM-cellulose (2 g/L); Tweens 20, 40, 60 and 80 (1%, v/v) and adenine, guanine, xanthine and hypoxanthine (0.5%, v/v). Anaerobic growth was determined in an anaerobic system (Anaero Pack-Micro Aero, 2.5-L, MGC, Japan) on ISP 2 supplemented with various electron acceptors as described by Chen, *et al*.^71^. Nitrate reduction was tested according to the protocol of Dong and Cai^72^. The methyl red and Voges-Proskauer tests were examined as described by Lányi^73^. Other biochemical properties and enzyme activities were tested using API ZYM and API 20NE kits (bioMérieux) according to the manufacturer’s instructions.

### Chemotaxonomic characterization

Cells used for the analysis of fatty acids were harvested from the third quadrants of ISP 2 agar plates. Fatty acid methyl esters (FAMEs) were extracted as described by Kuykendall, *et al*.^74^ and analyzed according to the instructions of the Microbial Identification System (MIDI; Microbial ID). Isoprenoid quinones were extracted using a CHCl_3_/MeOH mixture (2:1, v/v) from freeze-dried cells (500 mg) and analyzed using an HPLC-MS system (Agilent)^75^. Polar lipids were extracted and separated by two-dimensional thin-layer chromatography on silica gel 60 F_254_ plates (Merck). Molybdophosphoric acid, ninhydrin reagent, molybdenum blue, and α-naphthol/H_2_SO_4_ reagents were used for the detection of total lipids, lipids containing free aminolipids, phosphorus-containing lipids and glycolipids, respectively^76^. The analyses of sugars and amino acids in whole cell hydrolysates were performed following previous methods^77^.

### Preparation of the HM134^T^ fermented broth and extract

Strain HM134^T^ was inoculated into a 500-mL Erlenmeyer flask containing 200 mL of GYM medium (containing malt extract 10.0 g, yeast extract 4.0 g, glucose 4.0 g, CoCl_2_·6H_2_O 0.005 g in 1.0 L tap water at pH 7.0–7.2) as seed medium prior to fermentation process. Afterwards, 1% (v/v) of the starting stock culture was transferred to a 1 L Erlenmeyer flask containing 25% volume of the fermentation medium and incubated at 28 °C for 7 days on a rotary shaker at 250 rpm. The fermentation medium, H9A, consisted of soluble starch 20.0 g, glucose 20.0 g, soybean powder 10.0 g, yeast extract 5.0 g, malt extract 4.0 g, CaCO_3_ 2.0 g, MgSO_4_·7H_2_O 2.0 g, NaCl 3.0 g, in 1.0 l tap water at pH 7.0–7.2. All culture media were sterilized at 121 °C for 30 min. The cell-free supernatant was collected by centrifugation at 4,000 × rpm for 10 min then subjected to freeze drying process. The freeze-dried sample was repeatedly extracted with methanol and the final extract concentrated using a rotary evaporator at 40 °C. The final concentrate was suspended in dimethyl sulfoxide prior to bioactivity assays.

### *In vitro* anti-tumor cytotoxicity

HCT-116 (humancolorectal carcinoma), A549 (human lung carcinoma) and HepG2 (human hepatocellular carcinoma) cell lines were obtained from the Department of New Drug Screening, Zhejiang Hisun Pharmaceutical Co., Ltd.(Taizhou, China). Cell lines were maintained in Dulbecco’s modified Eagle’s medium supplemented with 10% (w/v) fetal bovine serum in a humidified incubator (5% CO_2_ in air at 37 °C). The antitumor activities of different concentrations of HM134^T^ extracts (20 and 100 μg/mL) were evaluated by the CCK-8 colorimetric method. The cell lines were cultured in DMEM containing 10% calf serum at 37 °C for 4 h in a 5% CO_2_ incubator. The adherent cells in the logarithmic growth phase were digested and seeded on a 96-well culture plate at a density of 1 × 10^4^ cells per/well. Test samples and controls were added to the medium and incubated for 48 h. Then, cell counting kit-8 (CCK-8, Dojindo) was added to the medium and incubated for 3 h. Cell viability was determined by measuring the absorbance at 450 nm using a SpectraMax M5 microplate reader (Molecular Devices Inc., Sunnyvale, CA, USA)^78^. The inhibitory rate of cell proliferation was expressed as IC_50_ values. Doxorubicin was used as a positive control while cells containing 0.5% DMSO were tested as negative control.

### Isolation and characterization of bioactive metabolites

The cultivation procedure described in section Preparation of the HM134^T^ Fermented Broth and Extract was repeated and the filtrate (30 L) of the culture broth was collected. The filtrate was separated and purified in an HP-20 macroporous resin (Mitsubishi, Japan) column, and then eluted with absolute ethyl alcohol. After concentrating to dryness using a rotary evaporator at 40 °C, the residue (30.0 g) was resolved by chromatography on a silica gel column eluted with n-heptane/ethyl acetate mixtures that were run with a growing polarity (100:0 to 30:70, v/v) to obtain six fractions (Fr1-6). Results of bioactivity assays (*in vitro* antitumor toxicity) indicated that the Fr1 (n-heptane/ethyl acetate, 95:5) fractions was cytotoxic *in vitro*. The active fraction was repeatedly purified, and separated on Sephadex LH-20 gel column (GE Healthcare, Glies, UK). Semi-preparative HPLC (Shimadzu LC-8A, Shimadzu-C18, 5 μm, 250×20 mm Shimadzu, Kyoto, Japan) were performed to obtain compounds **1** (8.4 mg).

Structural identification of the bioactive metabolite was conducted by spectroscopic analysis. ^1^H nuclear magnetic resonance (NMR) and ^13^C NMR spectra were acquired using a Bruker DRX-400 spectrometer (400 MHz for ^1^H and 100 MHz for ^13^C) (Bruker, Rheinstetten, Germany). Chemical shifts were reported in ppm. (δ). Residual CHCl_3_ (δ_H_ 7.26 ppm; δ_C_ 77.0) was used as an internal standard, with coupling constants (J) expressed in Hz. ^1^H and ^13^C NMR assignments were supported by the results of the ^1^H-1H COSY, HMQC, and HMBC experiments. The electrospray ionization MS data were recorded using a Time-of-Flight Mass Spectrometer X500R Q-TOF (Sciex, USA).

## Supplementary information

Supplementary Information.

Table S3.
