# Supplementary material for: Micromonospora zhangzhouensis sp. nov., a Novel Actinobacterium Isolated from Mangrove Soil, Exerts a Cytotoxic Activity in vitro
Source: Sci Rep. 2020 Mar 3;10:3889. doi: 10.1038/s41598-020-60677-0 (PMC7054543; doi:10.1038/s41598-020-60677-0)

Micromonospora zhangzhouensis sp. nov., a Novel Actinobacterium Isolated from Mangrove Soil, Exerts a Cytotoxic Activity *in vitro*

Geyi Fu[1], Ruijun Wang[1], Jinglin Ding[2], Huan Qi[3], Zhe Zhao[4], Can Chen[1], Hui Zhang[3,5], Zhenglian Xue[5], Jidong Wang[3,5]*, Min Wu[1,4]*

[1]Ocean College, Zhejiang University, Zhoushan, China

[2]Zhoushan Tourism & Health College, Zhoushan, China

[3]Zhejiang Key Laboratory of Antifungal Drugs, Zhejiang Hisun Pharmaceutical Co., Ltd., Taizhou, China

[4]College of Life Sciences, Zhejiang University, Hangzhou, China

[5]College of Biochemical Engineering, Anhui Polytechnic University, Wuhu, China

*** Correspondence:**
Jidong Wang (jdwang@hisunpharm.com) and Min Wu (wumin@zju.edu.cn).

Keywords: *Micromonospora* sp. nov., mangrove, polyphasic taxonomy, antitumor, bioactive metaboltes

Table S1 16s rRNA gene sequence similarities, Average nucleotide identity (ANI) value and GGDC dDDH value between strain HM134^T and related strains.

Strains	16s rRNA gene sequence similarity with HM134^T	Average nucleotide identity value with HM134^T	GGDC dDDH value with HM134^T (Formula 2)	Project accession number
M. rifamycinica CGMCC 4.2495^T	99.58	91.16	41.60	LT607752.1
M. wenchangensis CCTCC AA 2012002^T	99.44	95.21	62.60	GCA_002210435.1
M. krabiensis DSM 45344^T	99.03	82.85	25.70	LT598496.1
M. carbonacea DSM 43168^T	99.03	84.15	27.40	GCA_900091535.1
M. haikouensis DSM 45626^T	98.96	84.14	27.40	GCA_900091595.1
M. humi DSM 45647^T	98.75	83.30	26.50	GCA_900090105.1
M. coxensis DSM 45161^T	98.75	85.44	29.30	LT607753.1
M. matsumotoense DSM 44100^T	98.68	82.91	43.70	GCA_900091525.1
M. eburnean DSM 44814^T	98.54	82.91	26.90	GCA_900090225.1
M. marina DSM 45555^T	98.47	82.61	25.90	GCA_900091565.1

Table S2 Cellular fatty acid profile of strain HM134 and the type strains of related species

Strains: 1, HM134^T; 2, *M. rifamycinica* CGMCC 4.2495^T; 3, *M. wenchangensis* CCTCC AA 2012002^T; 4, *M. mangrovi* CCTCC AA 2012002^T.

Fatty acids that represented <0.5% in all strains are not shown, major fatty acids (>10%) in all strains are shown in bold. tr, trace (<0.5%); –, not detected. All data are from this study.

**Fatty acids**	1	2	3	4
**Saturated straight**				
$C_{10:0}$	tr	tr	1.0	3.9
$C_{14:0}$	1.0	0.8	1.0	-
$C_{16:0}$	3.0	3.0	3.4	2.5
$C_{17:0}$	1.2	1.1	0.9	-
$C_{18:0}$	5.2	2.2	5.7	8.2
**Saturated branched**				
iso-$C_{10:0}$	-	-	-	4.2
iso-$C_{14:0}$	3.0	1.5	2.2	4.4
iso-$C_{15:0}$	14.1	8.1	15.7	17.4
anteiso-$C_{15:0}$	2.2	5.1	2.4	7.9
iso-$C_{16:0}$	30.3	24.8	17.1	24.6
iso-$C_{17:0}$	3.2	2.5	4.2	6.1
anteiso-$C_{17:0}$	1.3	4.5	1.3	5.8
iso-$C_{18:0}$	0.6	tr	0.6	1.2
**Unsaturated**				
$C_{14:1}$ ω5c	tr	tr	0.5	1.0
iso-$C_{15:1}$ G	0.5	tr	1.5	-
$C_{15:1}$ ω8c	tr	-	0.6	-
iso-$C_{16:1}$ G	4.1	5.2	6.4	-
anteiso-$C_{17:1}$ A	tr	1.4	0.7	-
$C_{17:1}$ ω8c	1.5	3.9	0.7	-
$C_{18:1}$ ω9c	7.0	23.1	4.7	2.6
**Hydroxy**				
$C_{8:0}$ 3-OH	tr	tr	0.6	2.3
$C_{11:0}$ 2-OH	tr	tr	-	1.9
$C_{13:0}$ 2-OH	-	-	0.5	-
$C_{16:1}$ 2-OH	tr	-	0.6	-
**Methyl branched**				
10-methyl $C_{17:0}$	4.0	2.9	2.9	-
10-methyl $C_{18:0}$ (TBSA)	12.4	3.4	20.2	-
**Summed features**				
1*	-	-	-	2.2
3†	0.5	1.7	1.1	-
8‡	tr	0.5	tr	-
9 δ	2.7	2.5	3.1	3.7

*Summed feature 1 comprised *iso*-$C_{15:1}$ H and/or $C_{13:0}$ 3-OH.

†Summed feature 3 comprised $C_{16:1}$ ω7c and/or $C_{16:1}$ ω6c.

‡Summed feature 8 comprised $C_{18:1}$ ω7c or $C_{18:1}$ ω6c.

δ Summed feature 9 comprised *iso*-$C_{17:1}$ ω9c or 10-methyl $C_{16:0}$.

Table S4 ^{1}H (400 MHz) and ^{13}C (100 MHz) data of compound **1**

**No.**	**δ_H (*J* in Hz)**	**δ_C**
1	1.38(1H, m)	42.5d
2	2.46(1H, dd, 12.1, 2.9)	41.8d
3	6.91(1H, d, 12.4)	145.4d
4		128.4s
5	2.70(2H, m)	26.9t
6	3.21(1H, m) 2.34(1H, m)	28.1t
7	6.35(1H, t, 8.5)	145.4d
8		131.4s
9	4.11(1H, dd, 10.6, 4.6)	78.7d
10	1.96(1H, m) 1.64(1H, m)	39.3t
11	1.64(1H, m)	46.5d
12		150.0s
13	2.00(1H, m) 2.34(1H, m)	33.0t
14	1.22(1H, m) 1.77(1H, d, 12.4)	26.0t
15	1.22(1H, m)	30.9d
16	0.80(3H, d, 6.6)	21.5q
17	0.83(3H, d, 6.6)	21.0q
18		168.6s
19	3.75(3H, s)	51.8q
20	4.69(1H, br s) 4.64(1H, br s)	109.2t
21		170.0s

Table S5 Functional cluster of orthologous genes (COG) classification of predicted genes in strain HM134^T

**Code and Relative Description**	**No. of genes**	**percentage**
Information Storage and Processing		
[A] RNA processing and modification	1	0.02%
[J] Translation, ribosomal structure and biogenesis	277	5.39%
[K] Transcription	538	10.46%
[L] Replication, recombination and repair	166	3.23%
Cellular Process and Signaling		
[B] Chromatin structure and dynamics	3	0.06%
[D] Cell cycle control, cell division, chromosome partitioning	61	1.19%
[M] Cell wall/membrane/envelope biogenesis	256	4.98%
[N] Cell motility	28	0.54%
[O] Posttranslational modification, protein turnover, chaperones	211	4.10%
[T] Signal transduction mechanisms		
[U] Intracellular trafficking, secretion, and vesicular transport	45	0.87%
[V] Defense mechanisms	145	2.82%
[W] Extracellular structures	12	0.23%
[X] Mobilome: prophages, transposons	82	1.59%
[Z] Cytoskeleton	2	0.04%
Metabolism		
[C] Energy production and conversion	273	5.31%
[E] Amino acid transport and metabolism	429	8.34%
[F] Nucleotide transport and metabolism	120	2.33%
[G] Carbohydrate transport and metabolism	449	8.73%
[H] Coenzyme transport and metabolism	313	6.09%
[I] Lipid transport and metabolism	306	5.95%
[P] Inorganic ion transport and metabolism	298	5.79%
[Q] Secondary metabolites biosynthesis, transport and catabolism	270	5.25%
Poorly Characterized		
[R] General function prediction only	609	11.84%
[S] Function unknown	249	4.84%

Fig. S1 Maximum-parsimony phylogenetic tree based on the 16S rRNA gene sequences of strain HM134^T and representatives of related taxa. Bootstrap values are based on 1000 replicates; values above 50% are shown.

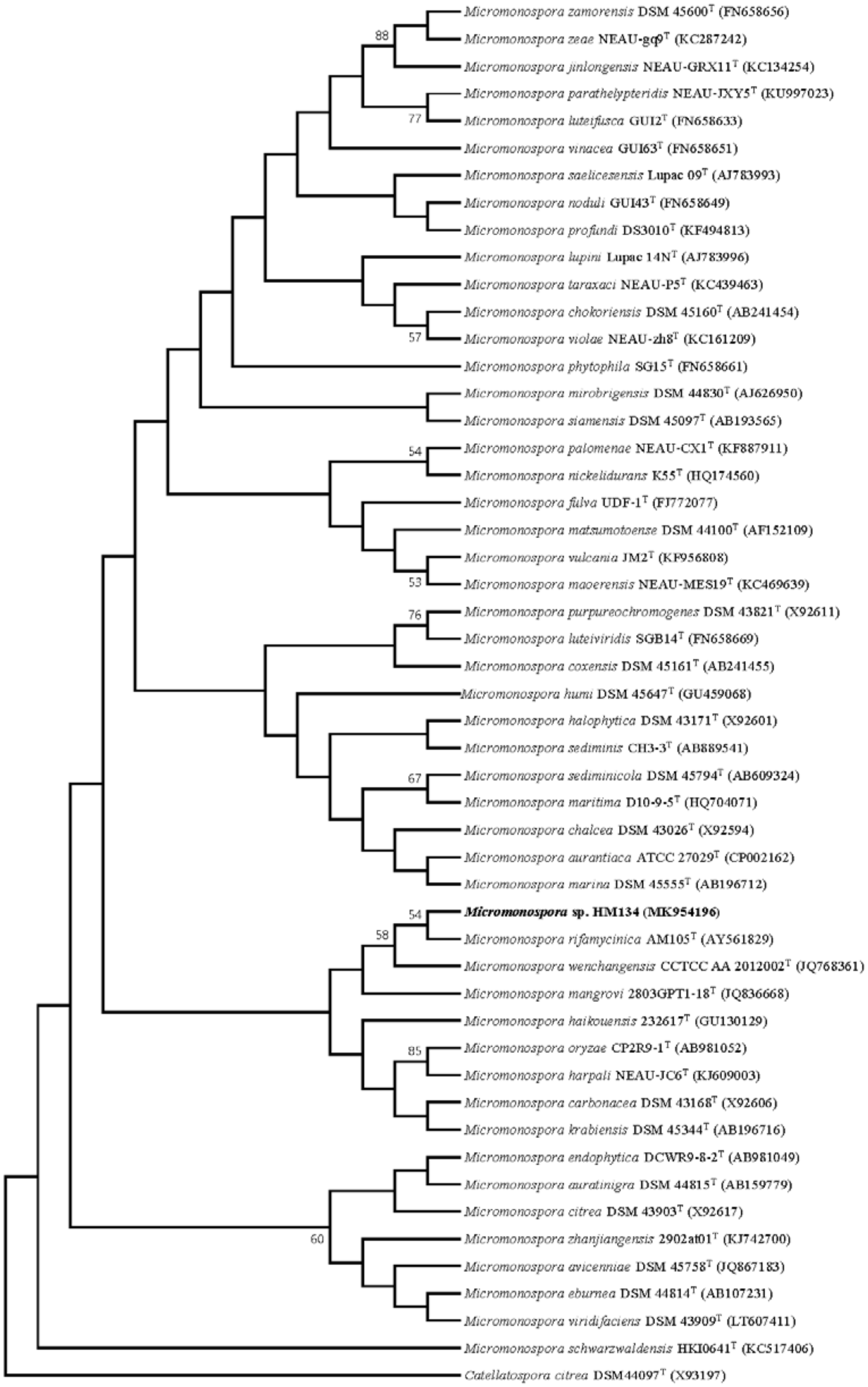

Fig. S2 Maximum-likelihood phylogenetic tree based on the 16S rRNA gene sequences of strain HM134^{T} and representatives of related taxa. Bootstrap values are based on 1000 replicates; values above 50% are shown. Bar, 0.005 substitutions per nucleotide position.

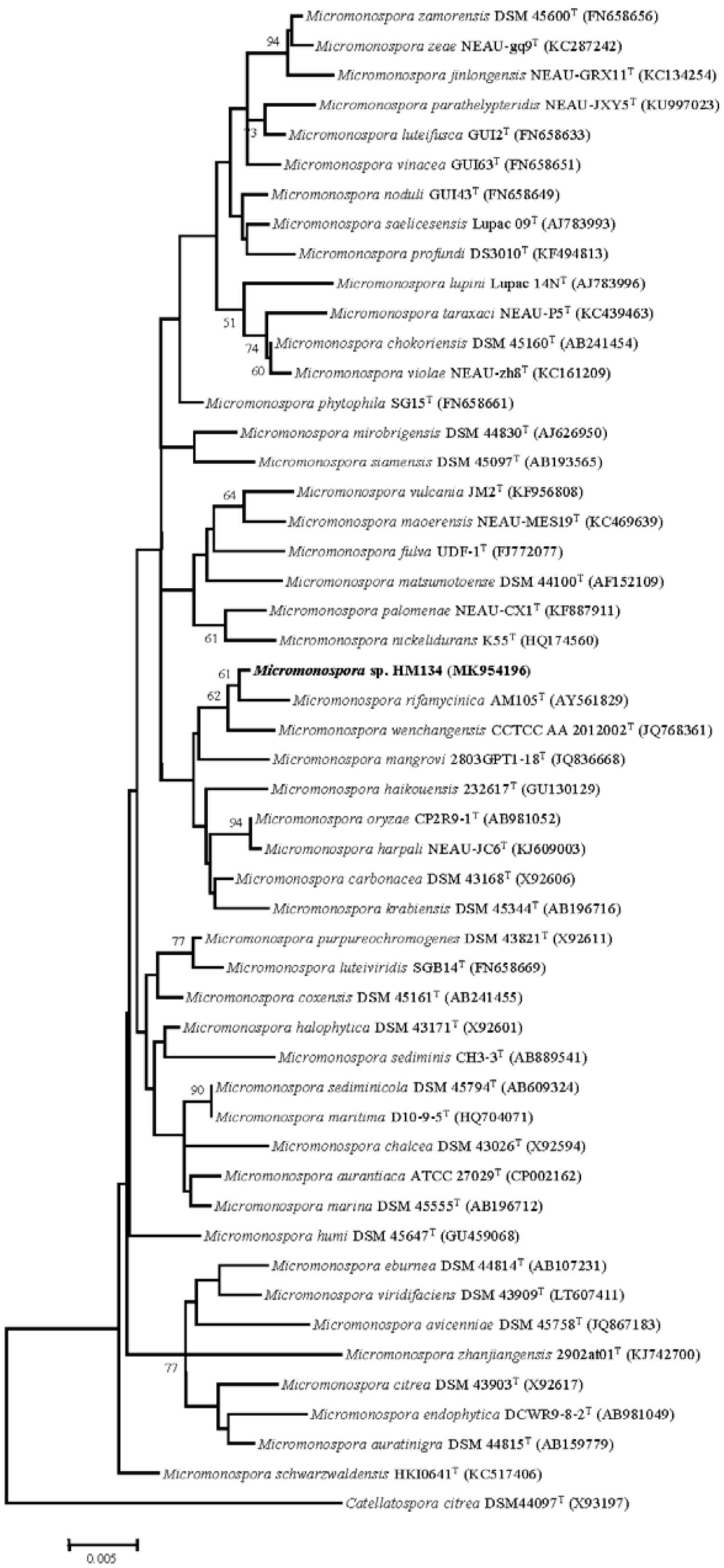

Fig. S3 Phylogenetic tree reconstructed by ARB software with database version arb-6.0.2, showing the relationship between 16S rRNA gene sequences from HM134^T and other species in *Micromonosporinea*. The scale bar represents 0.01 nucleotide change per position.

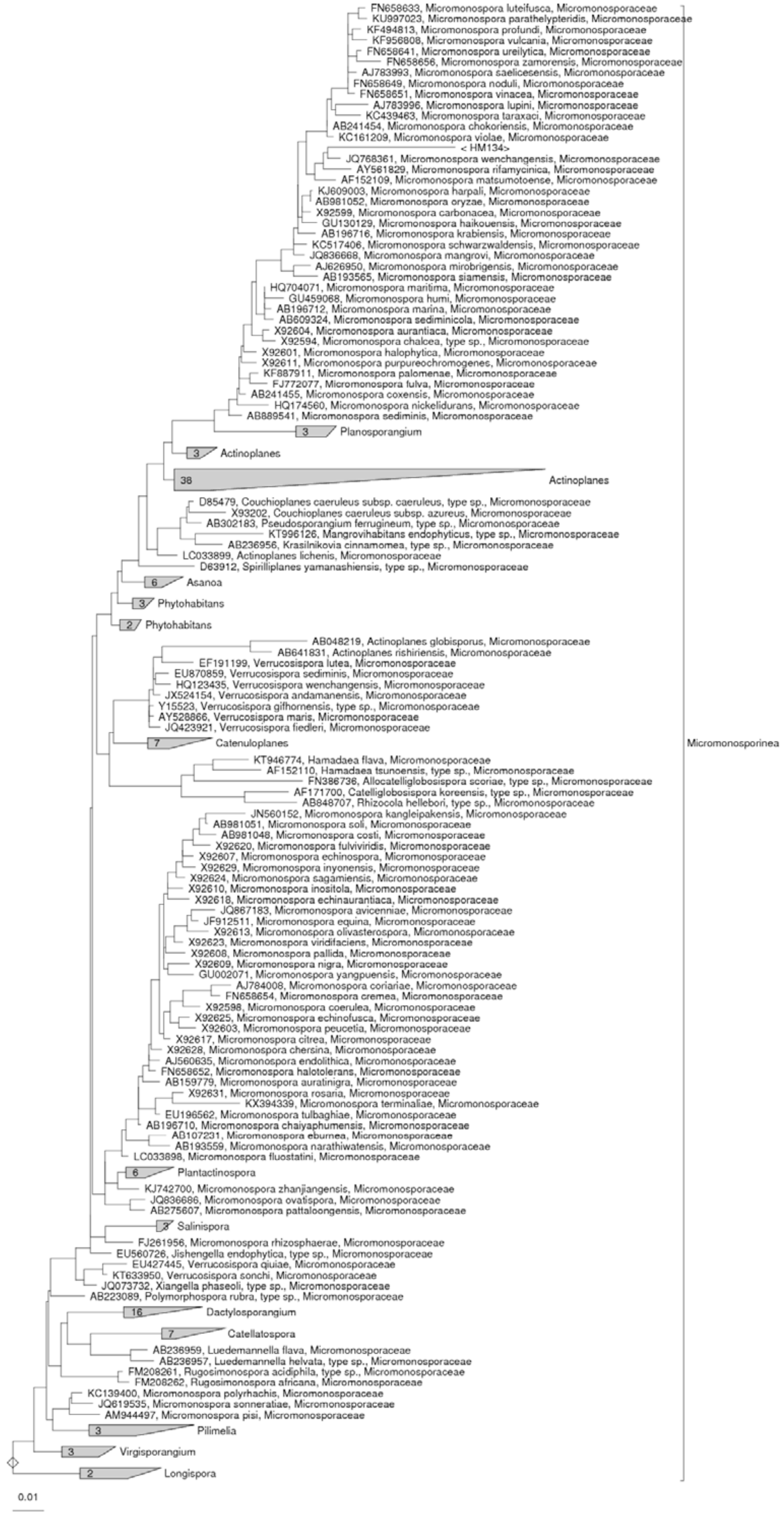

FN658633, Micromonospora luteifusca, Micromonosporaceae
KU997023, Micromonospora parathelypteridis, Micromonosporaceae
KF494813, Micromonospora profundi, Micromonosporaceae
KF956808, Micromonospora vulcania, Micromonosporaceae
FN658641, Micromonospora ureilytica, Micromonosporaceae
FN658656, Micromonospora zamorensis, Micromonosporaceae
AJ783993, Micromonospora saelicesensis, Micromonosporaceae
FN658649, Micromonospora noduli, Micromonosporaceae
FN658651, Micromonospora vinacea, Micromonosporaceae
AJ783996, Micromonospora lupini, Micromonosporaceae
KC439463, Micromonospora taraxaci, Micromonosporaceae
AB241454, Micromonospora chokoriensis, Micromonosporaceae
KC161209, Micromonospora violae, Micromonosporaceae
< HM134>
JQ768361, Micromonospora wenchangensis, Micromonosporaceae
AY561829, Micromonospora rifamycinica, Micromonosporaceae
AF152109, Micromonospora matsumotoense, Micromonosporaceae
KJ609003, Micromonospora harpali, Micromonosporaceae
AB981052, Micromonospora oryzae, Micromonosporaceae
X92599, Micromonospora carbonacea, Micromonosporaceae
GU130129, Micromonospora haikouensis, Micromonosporaceae
AB196716, Micromonospora krabiensis, Micromonosporaceae
KC517406, Micromonospora schwarzwaldensis, Micromonosporaceae
JQ836668, Micromonospora mangrovi, Micromonosporaceae
AJ626950, Micromonospora mirobrigensis, Micromonosporaceae
AB193565, Micromonospora siamensis, Micromonosporaceae
HQ704071, Micromonospora maritima, Micromonosporaceae
GU459068, Micromonospora humi, Micromonosporaceae
AB196712, Micromonospora marina, Micromonosporaceae
AB609324, Micromonospora sediminicola, Micromonosporaceae
X92604, Micromonospora aurantiaca, Micromonosporaceae
X92594, Micromonospora chalcea, type sp., Micromonosporaceae
X92601, Micromonospora halophytica, Micromonosporaceae
X92611, Micromonospora purpureochromogenes, Micromonosporaceae
KF887911, Micromonospora palomenae, Micromonosporaceae
FJ772077, Micromonospora fulva, Micromonosporaceae
AB241455, Micromonospora coxensis, Micromonosporaceae
HQ174560, Micromonospora nickelidurans, Micromonosporaceae
AB889541, Micromonospora sediminis, Micromonosporaceae
3 Planosporangium
3 Actinoplanes
38 Actinoplanes
D85479, Couchioplanes caeruleus subsp. caeruleus, type sp., Micromonosporaceae
X93202, Couchioplanes caeruleus subsp. azureus, Micromonosporaceae
AB302183, Pseudosporangium ferrugineum, type sp., Micromonosporaceae
KT996126, Mangrovihabitans endophyticus, type sp., Micromonosporaceae
AB236956, Krasilnikovia cinnamomea, type sp., Micromonosporaceae
LC033899, Actinoplanes lichenis, Micromonosporaceae
D63912, Spirilliplanes yamanashiensis, type sp., Micromonosporaceae
6 Asanoa
3 Phytohabitans
2 Phytohabitans
AB048219, Actinoplanes globisporus, Micromonosporaceae
AB641831, Actinoplanes rishiriensis, Micromonosporaceae
EF191199, Verrucosispora lutea, Micromonosporaceae
EU870859, Verrucosispora sediminis, Micromonosporaceae
HQ123435, Verrucosispora wenchangensis, Micromonosporaceae
JX524154, Verrucosispora andamanensis, Micromonosporaceae
Y15523, Verrucosispora gifhornensis, type sp., Micromonosporaceae
AY528866, Verrucosispora maris, Micromonosporaceae
JQ423921, Verrucosispora fiedleri, Micromonosporaceae
7 Catenuloplanes
Micromonosporinea
KT946774, Hamadaea flava, Micromonosporaceae
AF152110, Hamadaea tsunoensis, type sp., Micromonosporaceae
FN386736, Allocatelliglobosispora scoriae, type sp., Micromonosporaceae
AF171700, Catelliglobosispora koreensis, type sp., Micromonosporaceae
AB848707, Rhizocola hellebori, type sp., Micromonosporaceae
JN560152, Micromonospora kangleipakensis, Micromonosporaceae
AB981051, Micromonospora soli, Micromonosporaceae
AB981048, Micromonospora costi, Micromonosporaceae
X92620, Micromonospora fulviviridis, Micromonosporaceae
X92607, Micromonospora echinospora, Micromonosporaceae
X92629, Micromonospora inyonensis, Micromonosporaceae
X92624, Micromonospora sagamiensis, Micromonosporaceae
X92610, Micromonospora inositola, Micromonosporaceae
X92618, Micromonospora echinaurantiaca, Micromonosporaceae
JQ867183, Micromonospora avicenniae, Micromonosporaceae
JF912511, Micromonospora equina, Micromonosporaceae
X92613, Micromonospora olivasterospora, Micromonosporaceae
X92623, Micromonospora viridifaciens, Micromonosporaceae
X92608, Micromonospora pallida, Micromonosporaceae
X92609, Micromonospora nigra, Micromonosporaceae
GU002071, Micromonospora yangpuensis, Micromonosporaceae
AJ784008, Micromonospora coriariae, Micromonosporaceae
FN658654, Micromonospora cremea, Micromonosporaceae
X92598, Micromonospora coerulea, Micromonosporaceae
X92625, Micromonospora echinofusca, Micromonosporaceae
X92603, Micromonospora peucetia, Micromonosporaceae
X92617, Micromonospora citrea, Micromonosporaceae
X92628, Micromonospora chersina, Micromonosporaceae
AJ560635, Micromonospora endolithica, Micromonosporaceae
FN658652, Micromonospora halotolerans, Micromonosporaceae
AB159779, Micromonospora auratinigra, Micromonosporaceae
X92631, Micromonospora rosaria, Micromonosporaceae
KX394339, Micromonospora terminaliae, Micromonosporaceae
EU196562, Micromonospora tulbaghiae, Micromonosporaceae
AB196710, Micromonospora chaiyaphumensis, Micromonosporaceae
AB107231, Micromonospora eburnea, Micromonosporaceae
AB193559, Micromonospora narathiwatensis, Micromonosporaceae
LC033898, Micromonospora fluostatini, Micromonosporaceae
6 Plantactinospora
KJ742700, Micromonospora zhanjiangensis, Micromonosporaceae
JQ836686, Micromonospora ovatispora, Micromonosporaceae
AB275607, Micromonospora pattaloongensis, Micromonosporaceae
3 Salinispora
FJ261956, Micromonospora rhizosphaerae, Micromonosporaceae
EU560726, Jishengella endophytica, type sp., Micromonosporaceae
EU427445, Verrucosispora qiuiae, Micromonosporaceae
KT633950, Verrucosispora sonchi, Micromonosporaceae
JQ073732, Xiangella phaseoli, type sp., Micromonosporaceae
AB223089, Polymorphospora rubra, type sp., Micromonosporaceae
16 Dactylosporangium
7 Catellatospora
AB236959, Luedemannella flava, Micromonosporaceae
AB236957, Luedemannella helvata, type sp., Micromonosporaceae
FM208261, Rugosimonospora acidiphila, type sp., Micromonosporaceae
FM208262, Rugosimonospora africana, Micromonosporaceae
KC139400, Micromonospora polyrhachis, Micromonosporaceae
JQ619535, Micromonospora sonneratiae, Micromonosporaceae
AM944497, Micromonospora pisi, Micromonosporaceae
3 Pilimelia
3 Virgisporangium
2 Longispora
0.01

Fig. S4 Two-dimensional TLC of the polar lipids of strain HM134^T.
Spots were revealed by spraying the plates with 5% ethanolic molybdophosphoric acid. Diphosphatidylglycerol (DPG), phosphatidylethanolamine (PE), an unidentified phospholipid (PL1), three unidentified glycolipids (GL1, GL2 and GL3) and two unidentified lipids (L1 and L2).

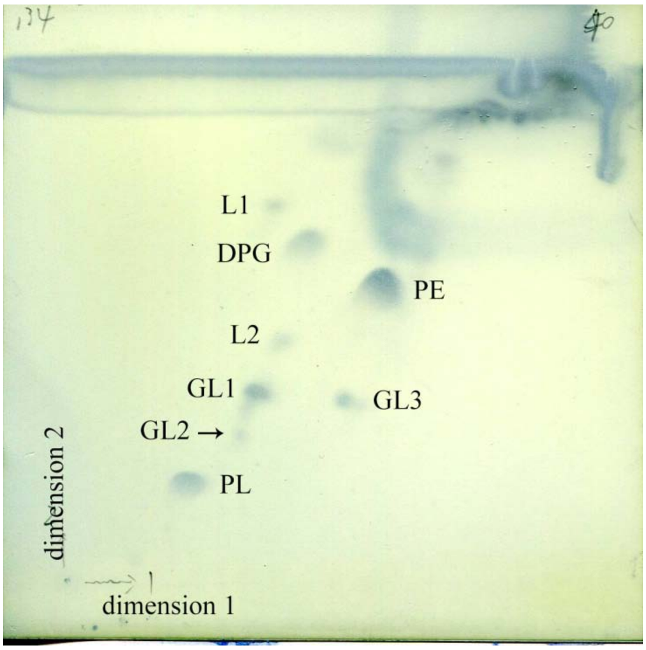

Fig. S5 Transmission electron micrograph (a) and scanning electron micrographs (b) of substrate mycelium of culture of strain HM134^T after growth on ISP 2 agar for 21 days at 28 °C. Bar, 5 μm(a) and 2 μm(b).

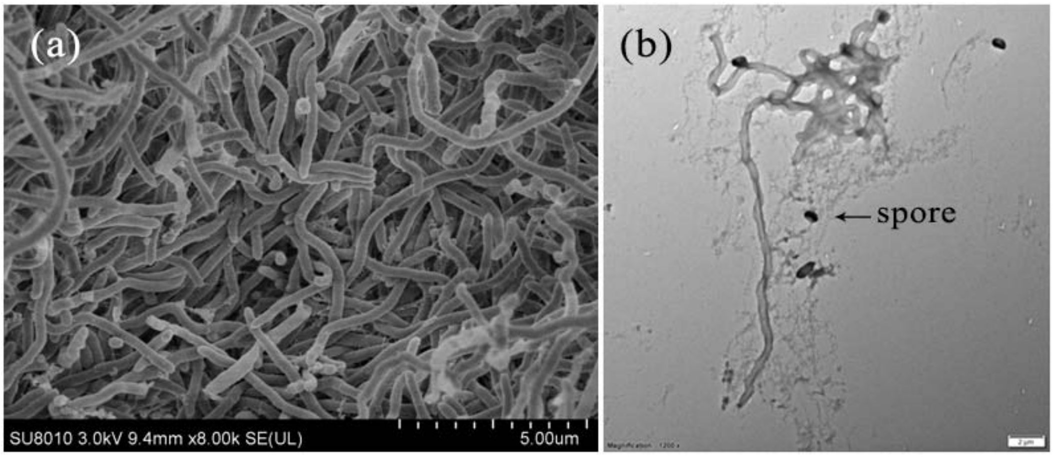

Fig. S6 TOF-MS of Compound 1 in MeOH

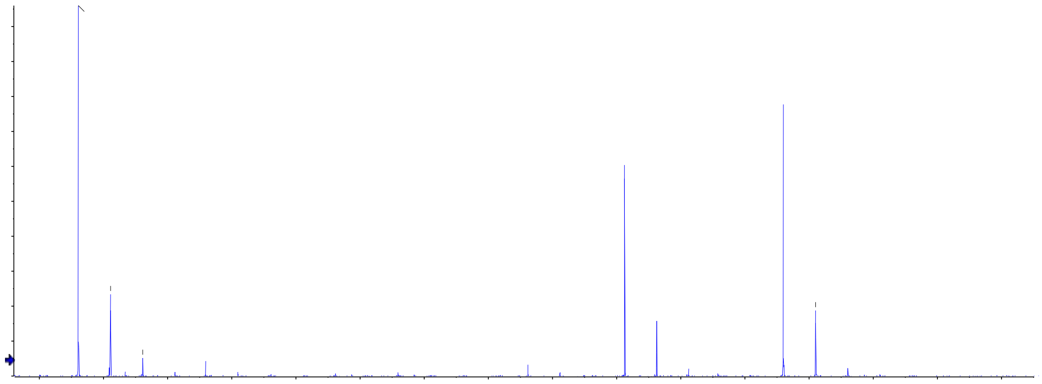

Fig. S7 IR spectrum of Compound 1

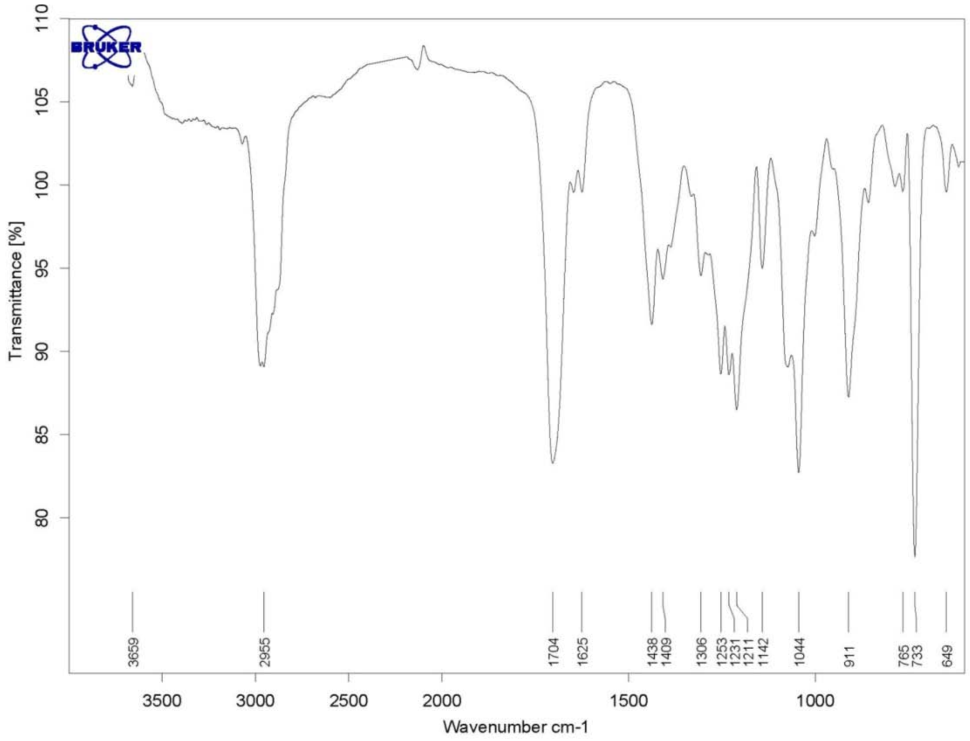

Fig. S8 ^{1}H NMRspectrum (400 MHz) of Compound 1in $CDCl_3$

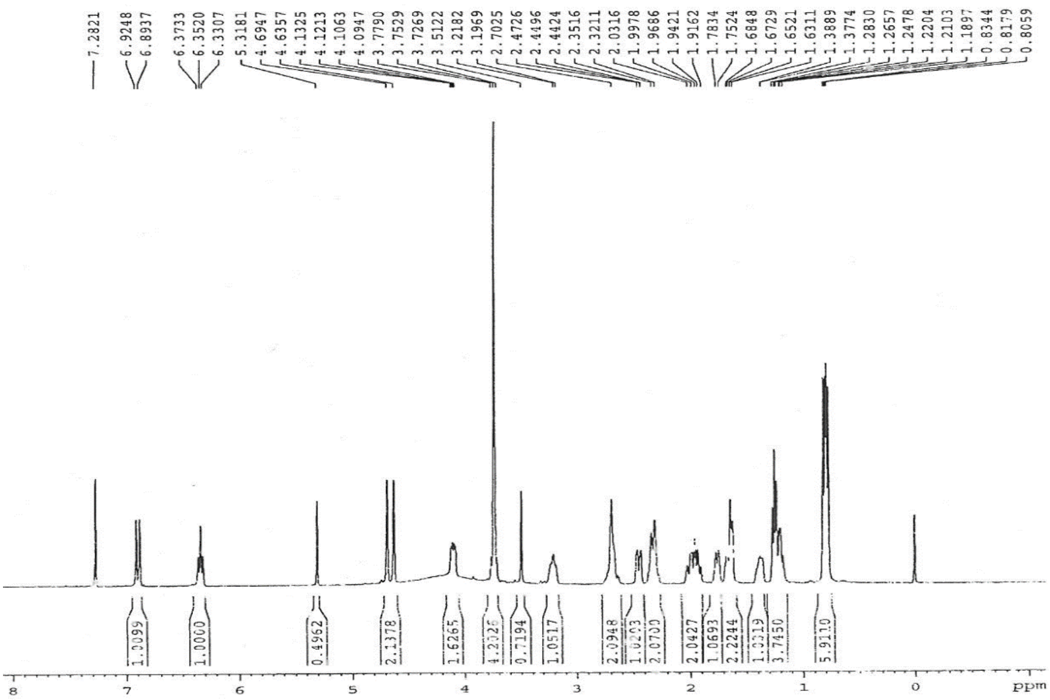

Fig. S9 ^{1}H NMRspectrum (400 MHz) of Compound 1in $CDCl_3$

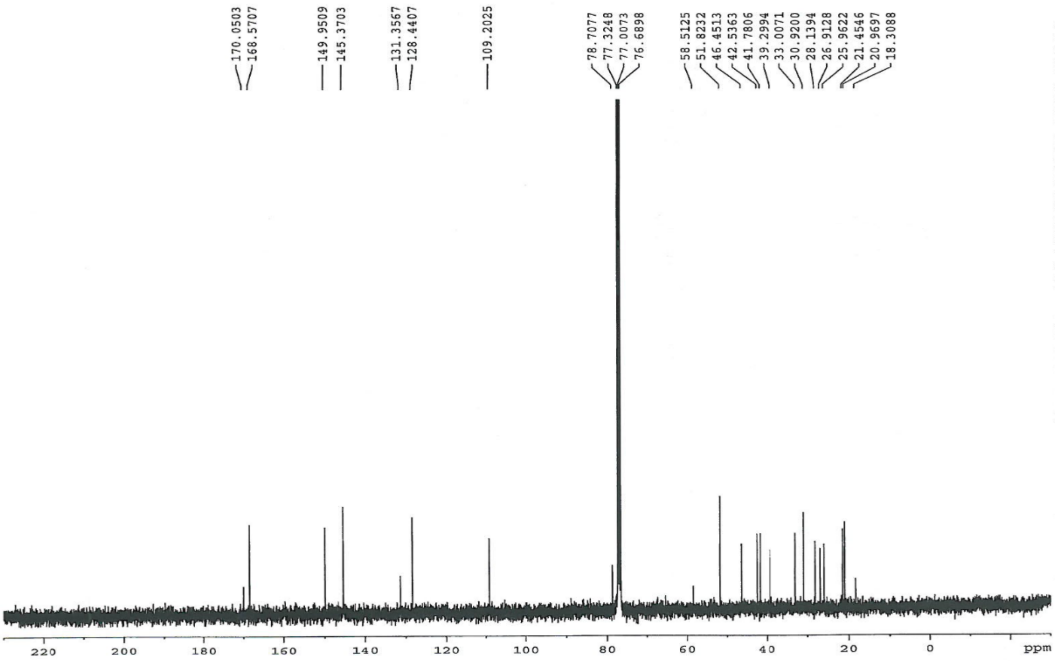

Fig. S10 DEPT135 spectrum (100 MHz) of Compound 1in $CDCl_3$

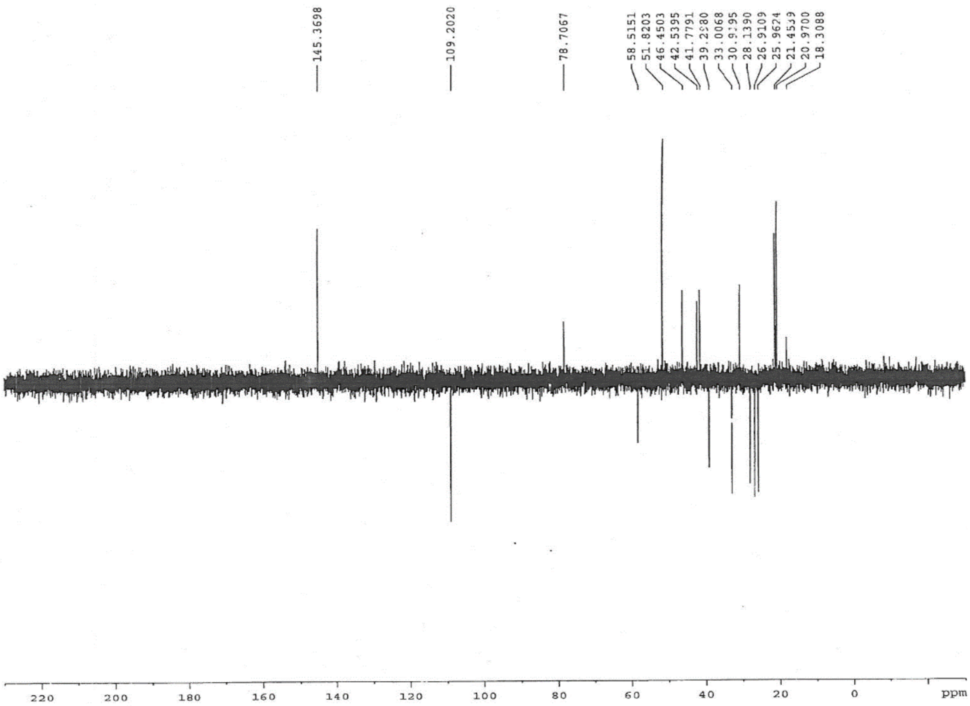

Fig. S11 The ^1H-^1H COSY spectrum (400 MHz) of Compound 1in $CDCl_3$

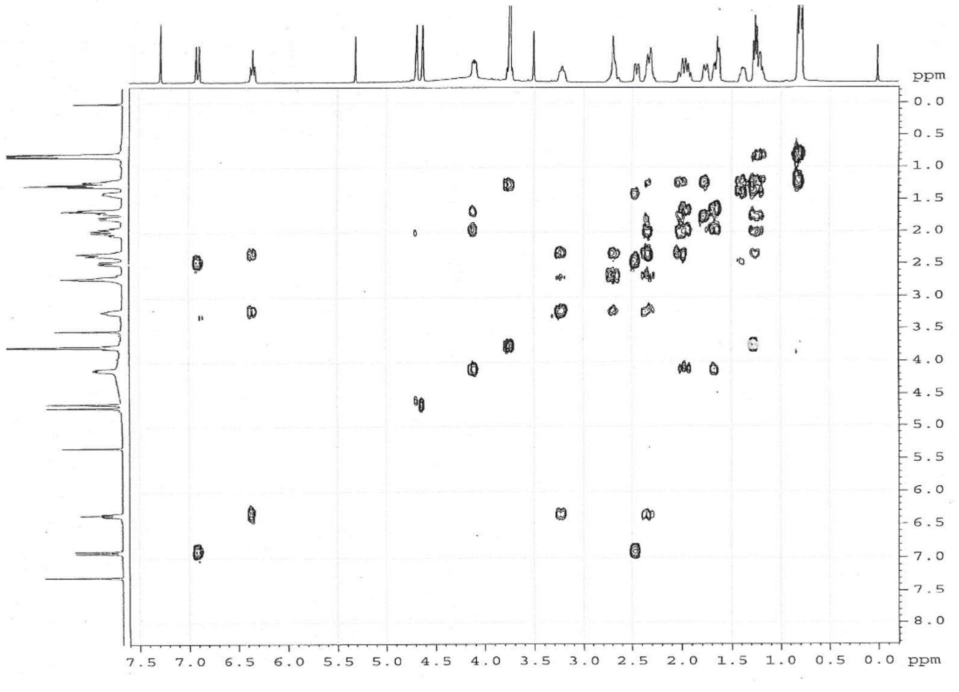

Fig. S12 The HSQC spectrum of Compound 1in $CDCl_3$

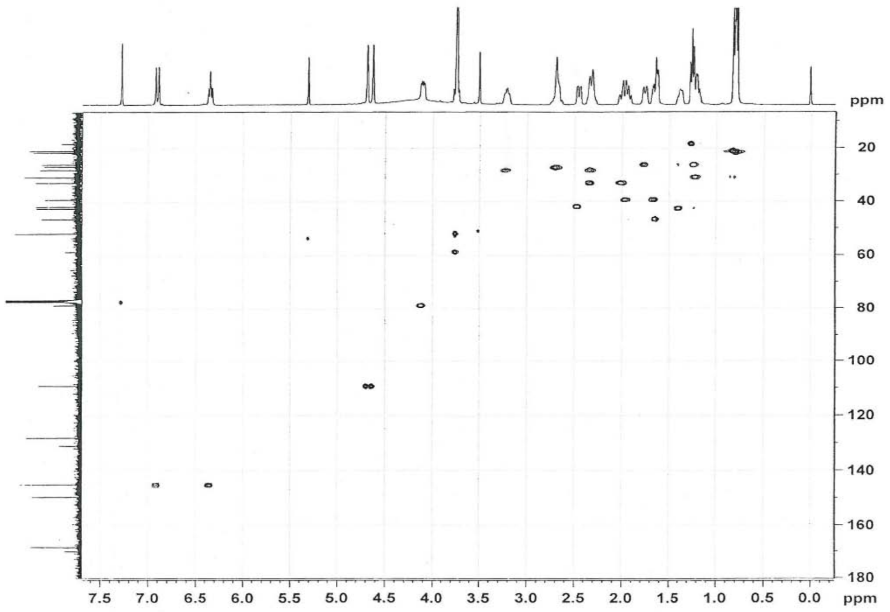

Fig. S13 The HMBC spectrum of Compound 1 in $CDCl_3$

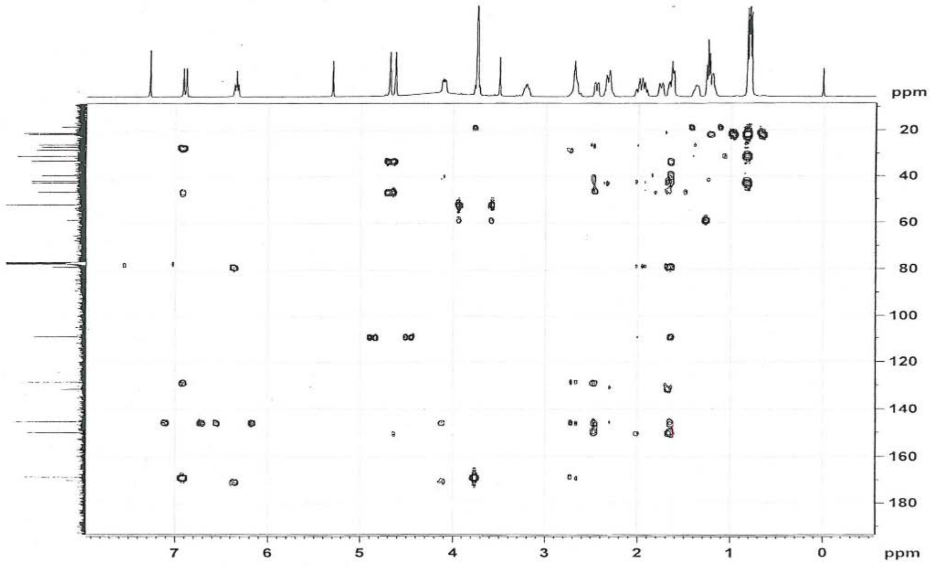

Fig. S14 The NOESY spectrum of Compound 1 in $CDCl_3$

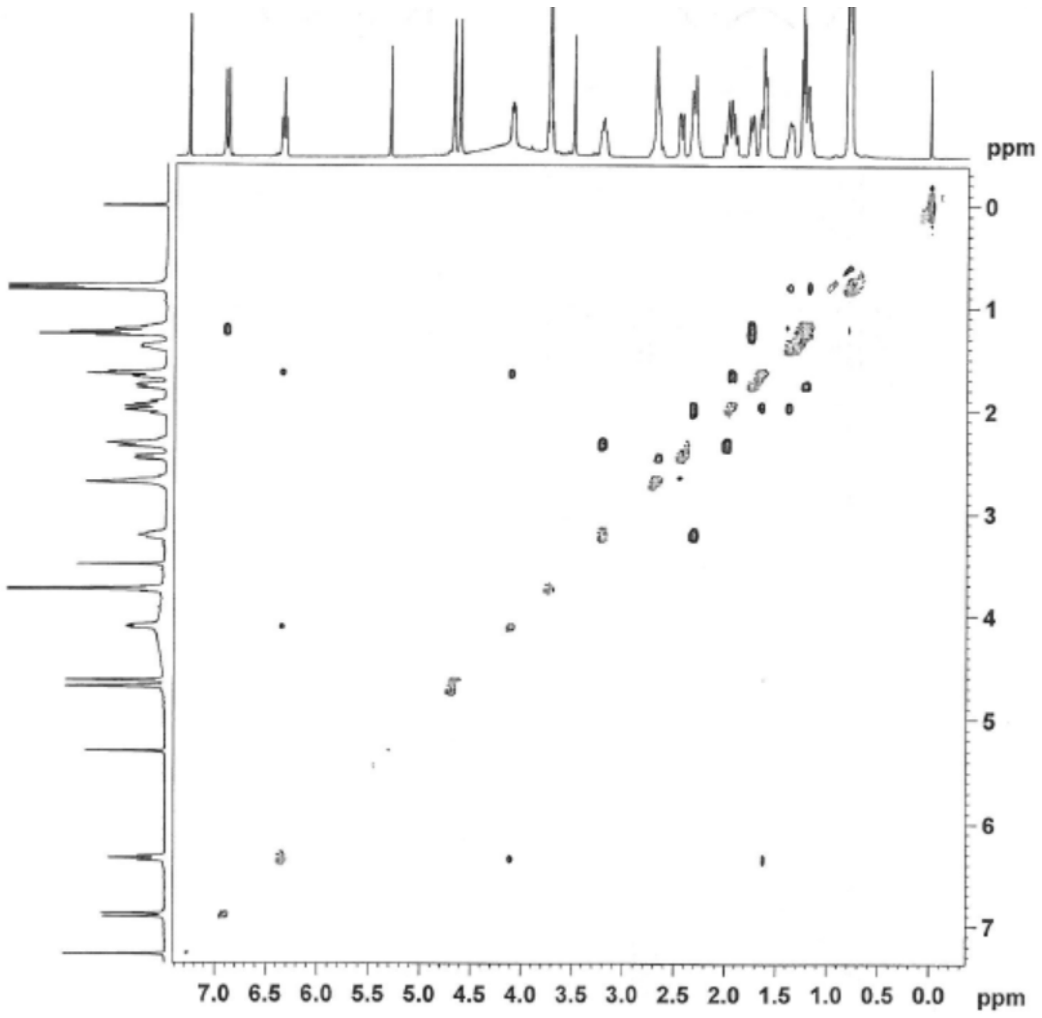

Supplement: Supplementary file 1 — Supplementary Information. [file 41598_2020_60677_MOESM1_ESM.pdf]
